# Evaluation of outcomes of medication therapy management (MTM) services for patients with chronic obstructive pulmonary disease (COPD)

**DOI:** 10.12669/pjms.37.7.4518

**Published:** 2021

**Authors:** Mingyue Liu, Jiayun Liu, Zhihui Geng, Shuang Bai

**Affiliations:** 1 Mingyue Liu, Department of Pharmacy, Baoding No.1 Central Hospital, Baoding, Hebei, 071000, China; 2 Jiayun Liu, Department of Anesthesiology, Baoding No.1 Central Hospital, Baoding, Hebei, 071000, China; 3 Zhihui Geng, Department of Pharmacy, Baoding No.1 Central Hospital, Baoding, Hebei, 071000, China; 4 Shuang Bai Department of Pharmacy, Baoding No.1 Central Hospital, Baoding, Hebei, 071000, China

**Keywords:** Medication therapy management (MTM), chronic obstructive pulmonary disease (COPD), outcome

## Abstract

**Objectives:**

To evaluate the outcomes of clinical pharmacist-led medication therapy management (MTM) services for patients with chronic obstructive pulmonary disease (COPD).

**Methods:**

Two hundred COPD patients admitted by the Department of Respiratory and Critical Care Medicine of Baoding No.1 Central Hospital during January 2019 and December 2020 were randomly assigned to a control group (n =100) and an experimental group (n =100). Patients in the control group received conventional treatment, while those in the experimental group were provided with MTM services based on the conventional treatment for comparative analysis of outcome measures, including use of antibacterials during hospital stay, length of stay (LoS), costs of hospitalization (CoH), cases of adverse drug reactions (ADRs), and medication adherence (MA) and COPD assessment test (CAT) score one and six months after discharge.

**Results:**

Compared with the control group, the experimental group had reduced use of antibacterials during hospital stay, LoS, CoH, and ADR rate (P <0.05). After discharge, patients in both groups showed remarkable improvements in MA and CAT scores in comparison with their performances upon admission, and the experimental group exhibited better MA and higher CAT score than the control group, with the differences indicating statistical significance (P <0.05).

**Conclusion:**

MTM designed for COPD patients can improve pharmacist-led service quality and clinical outcomes of COPD.

## INTRODUCTION

Chronic obstructive pulmonary disease (COPD) is a common, preventable and treatable condition characterized by persistent respiratory symptoms and airflow limitation.^1^ As the prevalence keeps climbing worldwide every year, COPD has become one of the most commonly seen chronic diseases resulting in a heavy social and economic burden to the global community.^2^ Old age, comorbidities, multidrug therapy, and poor inspiratory capacity often cause a variety of drug-related problems (DRPs) in COPD patients.^3^ Standard medication and treatment guidelines, effective assessment of patient understanding of treatment plans, and regular follow-ups are beneficial to patient medication adherence (MA) and therapeutic efficacy by preventing misuse of drugs, reducing medical costs and facilitating rational therapy.^4^

Medication therapy management (MTM) refers to a group of standard services provided by clinical pharmacists, such as pharmacy consulting, medication monitoring, and medication education for patients, to improve MA, address DRPs in COPD cases, optimize therapeutic outcomes and ultimately achieve effective disease management with patients.^5^ In this study, the Hepler–Strand classification system^6^ was employed to classify and analyze DRPs in COPD from the aspects of indications, effectiveness, safety, and MA^7^, aiming to provide strategic guidance for the hospital to improve pharmaceutical care for COPD patients.

## METHODS

### Ethical approval

The study was approved by the Institutional Ethics Committee

A total of 200 COPD patients who were hospitalized at the Department of Respiratory Medicine between January 1, 2019 and December 31, 2020 were enrolled in this study and randomly divided into two groups (control vs trial) of 100 patients. Except for seven patients who were lost to follow up, the rest 193 patients had completed this study, including 97 from the control group and 96 from the experimental group. The study design has obtained approval from the hospital’s ethical committee of Baoding No.1 Central Hospital, and written informed consent was obtained from all participants. (April 12,2021).

### Inclusion criteria

A patient was eligible for this study if he/she was a) over 40 years old, b) treated by the Department of Respiratory and Critical Care Medicine for COPD, c) met the COPD diagnostic criteria given in the Global Initiative for Chronic Obstructive Lung Disease (GOLD) guidelines and free of hearing defect, disorders of speech and language or cognitive impairment, d) voluntary to participate in this study with informed consent, and e) able to maintain good communication with researchers to complete this study as required.

### Exclusion criteria

A patient was rendered ineligible if he/she a) died during the study, b) registered with invalid contact information, c) showed a lack of cooperation during the follow-up period, d) had cognitive impairment, or e) expressed unwillingness or refusal to cooperate.

### Methods

The control group was treated with conventional medication, including antibacterials, bronchodilators, inhaled hormones, and expectorants. The experimental group was provided with MTM services combined with the conventional treatment. The MTM services mainly included establishing a clinical database for each patient in 1-3 days from the date of admission, undertaking a CAT questionnaire (a validated COPD-specific questionnaire for assessing the impact of COPD on quality of life) survey, medication education for COPD patients, and patient MA survey during hospital stay. A retrospective analysis of each patient’s prior use of medication was conducted to evaluate the therapeutic outcomes and make necessary adjustments to the prescription. Adverse drug events and misuse of drugs were recorded and arranged in the order of severity to identify DRPs and develop a problem-solving plan. Two follow-up surveys were carried out 1 and 6 months after discharge to learn about self-administering medication at home, CAT score, and patient MA.

The outcome measures chosen for comparative analysis were post-treatment antibacterial use, LoS, CoH, ADRs, patient MA, and CAT score.

Defined daily doses (DDDs) of antibacterials, antibacterial usage rates, and combined modality rates in the two groups were compared to assess rational use of antibacterials in COPD patients according to the Guiding Principles of Clinical Use of Antibiotics (2015, China), Expert Consensus on Anti-infective Therapy for Acute Exacerbation of Chronic Obstructive Pulmonary Disease^8^, and the hospital’s administrative rules for clinical use of antibacterials.

LoS, CoH, and ADR rate: (1)LoS and CoH: Mean LoS and CoH were calculated for intergroup comparison. (2)Frequencies and details of ADRs during hospitalization and the follow-up period were recorded and assessed using the Naranjo ADR Probability Scale.^9^ On this basis, ADRs classified as “possible”, “probable”, and “definite” were utilized to determine the rate of ADRs in each group with the following formula: Rate of ADRs = Frequency of ADRs / total number of patients ×100%.

### MA and CAT scores: (1) MA score

The 8-question version of the Morisky Medication Adherence Scale (MMAS-8) was used for MA assessment.^10^ MMAS-8 consists of 8 questions, and total scores range from 0 to 8. Patient MA is considered high if the score is 8, medium if 6 to <8, and low if <6. MA was assessed upon admission and one and six months after discharge to investigate variations in the two groups’ MA scores. ***(2) CAT score:*** The CAT was employed to assess the impact of COPD on quality of life. Total CAT scores can range from 0-40 and have been categorized into four levels: low impact (score: 0-10), moderate impact (score: 11-20), high impact (score: 21-30), and very high impact (score: 31-40). Assessments were carried out upon admission and one and six months after discharge for comparative analysis.

### Data processing

The software SPSS 19.0 was used for statistical analysis. Measurement data were expressed by “mean ± standard deviation (x±s)”, and enumeration data were represented as percentage (%). The t-test and the χ2 test were employed to evaluate the study results. Significance was established at the level of P <0.05.`

## RESULTS

A total of 193 had completed this study, including 97 from the control group and 96 from the experimental group. No significant differences in demographic characteristics were observed between the two groups (P >0.05), suggesting a high degree of comparability.Table-I. The DDDs of antibacterials, antibacterial usage rate, and combined modality rate in the experimental group were significantly lower than in the control group (P <0.05). Table-II. The LoS, CoH, and ADR rate in the experimental group were significantly lower than in the control group (P <0.05). Table-III.

**Table I:** Comparison of demographic characteristics between the control and experimental groups [n(%), x±s].

*Item*		*Control (n =97)*	*Experimental (n =96)*	*P*
Sex	Male	63(64.95)	66(68.75)	>0.05
	Female	34(35.05)	30(31.25)	>0.05
Mean age (yrs)		73.25±7.45	75.13±8.03	>0.05
Smoking history	Yes	59(60.82)	57(59.38)	>0.05
	No	38(39.18)	39(40.63)	>0.05
Comorbid chronic conditions	Yes	73(75.26)	75(78.13)	>0.05
	No	24(49.48)	21(21.88)	>0.05

**Table II:** Comparison of use of antibacterials between the control and experimental groups (x±s).

*Item*	*Control (n =97)*	*Experimental (n =96)*	*P*
DDDs	189	121	<0.05
Antibacterial usage rate[n(%)]	93(95.88)	71(73.96)	<0.05
Combined modality rate[n(%)]	73(75.26)	23(23.96)	<0.01

**Table III:** Comparison of LoS, CoH, and ADR rate (x±s).

*Item*	*Control (n =97)*	*Experimental (n =96)*	*P*
LoS (d)	13.46±3.95	11.27±5.28	<0.05
CoH (RMB)	14,856.51±4,521.63	13,405.45±4,023.78	<0.05
ADR rate[n(%)]	23(23.71)	9(9.38)	<0.01

Upon admission, there were no significant differences in MA and CAT scores between the two groups (P >0.05). Both groups showed visible improvements in MA and CAT scores 1 and 6 months after discharge compared with their performances upon admission (P <0.05), and the experimental group markedly outperformed the control group in MA and CAT scores 1 and 6 months after discharge (P <0.05). Table-IV.

**Table IV:** Comparison of MA and CAT scores upon admission and 1 and 6 months after discharge (x±s, pts).

*Group (n)*	*Period*	*MA score*	*CAT score*
Control (n =97)	Upon admission	5.28±0.43	28.47±1.59
	1 month after discharge	6.12±0.41^a^	13.61±2.23^a^
	6 months after discharge	6.05±0.39^a^	15.61±2.01^a^
Experimental (n =96)	Upon admission	5.24±0.49	29.31±1.71
	1 month after discharge	7.45±0.37^ab^	8.73±1.69^ab^
	6 months after discharge	7.31±0.46^ab^	9.03±1.75^ab^

***Note:***

a P <0.05 compared with MA and CAT scores upon admission;

b P<0.05 compared with the control group 1 and 6 months after discharge.

## DISCUSSION

COPD is a common, frequently-occurring disease among the elderly population. With the aging population growing, the incidence of COPD keeps increasing every year in China. Among the approximately 40 million COPD patients in China, the prevalence of COPD among adults over 40 years old is up to 9.9%. The chronic condition has an adverse impact on each patient’s quality of life^11^ and predisposes the respiratory system to DRPs during the course of treatment.^12^ Foreign studies show that COPD in about 30% of the patients is associated with other underlying conditions, and over 68% of the elderly patients have to take five or more drugs on a daily basis.^13^ Combined-modality treatment often leads to misuse of drugs and other DRPs. It is shown that when adding one more drug to the regimen, the risk of DRPs is increased by 10%, and the patient becomes at higher risk of getting hospitalized.^14^,^15^ As conventional COPD treatment does not include pharmacist-led pharmaceutical care, it is difficult to achieve desired therapeutic outcomes with the resultant poor MA (regarding the proper use of inhaled drugs, and long-term, regular medication), consequently increasing COPD exacerbations and the frequency of hospitalization every year^16^,^17^.

Pharmacist-led MTM services primarily include five core elements: medication therapy review, personal medication record, medication-related action plan, intervention and/or referral, and documentation and follow-up.^18^ A standardized pharmaceutical care program to encourage active MTM among patients with COPD is expected to improve patient MA and medication safety at home, prevent misuse of drugs, reduce ADRs, promote rational use of drugs in clinical practice and help patients reduce medical costs.^19^-^21^

In this study, 193 patients who were randomized into a control group and an experimental group had completed the trial. Clinical pharmacist-led MTM combined with conventional treatment was implemented in the experimental group. Compared with the control group, the experimental group exhibited substantially reduced in-hospital DDDs of antibacterials, antibacterial usage rate, combined modality rate, LoS, CoH, and ADR rate, with the differences between the two groups showing statistical significance (P <0.05). Besides, both groups presented visible improvements in MA and CAT scores 1 and 6 months after discharge when compared with their performances upon admission, and the differences were significant (P <0.05). Thanks to standard medication education and regular follow-ups, the experimental group outperformed the control group in MA and CAT scores, suggesting differences at a significant level (P <0.05). The study results showed that MTM services could promote rational use of antibacterials in clinical practice, improve the therapeutic efficacy of the medication and reduce medical costs. However, the scope of this study is limited to a Grade III, Level A hospital in a given time frame.

In summary, the aging population in China is predisposed to COPD, one of the most common chronic conditions. In clinical diagnosis and treatment, patient awareness and MA can directly influence therapeutic outcomes. MTM services combined with domestic and foreign medication concepts are beneficial to the treatment for hospitalized patients with COPD, as well as safe, efficient, and cost-effective use of medication; MTM also helps reduce LoS, CoH, and ADR rate and improve patient MA and control over COPD. Therefore, MTM services are worthy of promotion in clinical practice as an innovative pharmacist-led pharmaceutical care model.

### Authors’ Contributions:

**ML &** **JL:** Designed this study and prepared this manuscript,and are responsible and accountable for the accuracy or integrity of the work.

**ZG:** Collected and analyzed clinical data.

**SB:** Significant contribution in revision of manuscript.
